# Cellular Complexity in MAPK Signaling in Plants: Questions and Emerging Tools to Answer Them

**DOI:** 10.3389/fpls.2018.01674

**Published:** 2018-11-27

**Authors:** Patrick J. Krysan, Jean Colcombet

**Affiliations:** ^1^Horticulture Department, University of Wisconsin–Madison, Madison, WI, United States; ^2^Institute of Plant Sciences Paris Saclay (IPS2), CNRS, INRA, Université Paris-Sud, Université d’Evry, Université Paris-Saclay, Gif-sur-Yvette, France; ^3^Institute of Plant Sciences Paris Saclay (IPS2), CNRS, INRA, Université Paris-Sud, Université d’Evry, Université Paris-Diderot, Sorbonne Paris-Cité, Gif-sur-Yvette, France

**Keywords:** signaling cascade, MAPK, phosphorylation, plant, microscopy, activity sensors

## Abstract

Mitogen activated protein kinase (MAPK) cascades play an important role in many aspects of plant growth, development, and environmental response. Because of their central role in many important processes, MAPKs have been extensively studied using biochemical and genetic approaches. This work has allowed for the identification of the MAPK genes and proteins involved in a number of different signaling pathways. Less well developed, however, is our understanding of how MAPK cascades and their corresponding signaling pathways are organized at subcellular levels. In this review, we will provide an overview of plant MAPK signaling, including a discussion of what is known about cellular mechanisms for achieving signaling specificity. Then we will explore what is currently known about the subcellular localization of MAPK proteins in resting conditions and after pathway activation. Finally, we will discuss a number of new experimental methods that have not been widely deployed in plants that have the potential to provide a deeper understanding of the spatial and temporal dynamics of MAPK signaling.

## Introduction

Signal transduction refers to the process by which information flows through a living system. At the molecular level, this flow of information is accomplished by the movement of signaling molecules within a cell. In order to fully understand a signaling pathway, it is therefore important to characterize the spatial and temporal dynamics of the different components of that pathway. In the case of mitogen activated protein kinase (MAPK) signaling, critical factors to consider include the subcellular localizations of the kinases, the interaction partners of the kinases and how these factors change in space and time when the pathways are activated and deactivated. In this review, we discuss what is currently known about the subcellular localizations and movements of MAPK cascade proteins in plants and highlight new approaches that have the potential to improve the depth of our understanding of this largely unexplored aspect of plant MAPK signaling.

## Signaling Processes in MAPK Modules

### Plant Genomes Code for Many Actors of MAPK Modules

MAPK (for Mitogen-Activated Protein Kinase) modules are found in all eukaryotic organisms, from fungi -they are particularly well studied in *Saccharomyces cerevisiae*-, to mammals and plants, and they are remarkably conserved in terms of structure and organization in modules. A MAPK module, also often referred as a MAPK cascade, is minimally constituted of three kinases activating each other by phosphorylation of their activation loops in a serial way: A MAPK is activated by a MAPK Kinase (or MAP2K) which is itself activated by a MAP2K Kinase (or MAP3K). The *Arabidopsis thaliana* genome contains 20 *MAPK* and 10 *MAP2K* genes (Ichimura et al., 2002; Colcombet and Hirt, 2008). Under the MAP3K label, several plant families have been gathered by homology with animal MAP3Ks. The best known group is the MAPK/ERK Kinase Kinase (MEKK)-like MAP3K family which has 20 members and has been repeatedly shown to be indeed able to activate MAP2Ks. The Raf and ZIK kinases constitute 48 and 11 member families which, despite having been involved in various physiological processes, have rarely been unambiguously shown to act as MAP2K activators. To the contrary, some Rafs seem to be negative regulators of MAPK modules (Yoo et al., 2008; Zhao et al., 2014). This review will be focused on the MEKK-like MAP3K family since this group has been most clearly shown to function as canonical MAP3Ks. Just considering combinatorial modules constituted of a MAPK, a MAP2K and a MAP3K of the MEKK family, there are potentially a very large number of putative functional MAPK modules in plant cells. The upstream steps involved in the activation of MAPK modules remain largely unclear. Recent works involving kinases of the Receptor-Like Cytosolic Kinase (RLCK) family has shown that these kinases might connect plasma membrane Pattern Recognition Receptors (PPRs) to the activation of intracellular MAPK modules by elicitors (Yamada et al., 2016; Wang et al., 2017; Bi et al., 2018; Rao et al., 2018; Sun et al., 2018; Yan et al., 2018). Additionally, plant genomes code for kinases with homology to yeast Ste20 kinase which, in some cases, act as MAP3K Kinases (MAP4Ks) (Dan et al., 2001; Boyce and Andrianopoulos, 2011). Their functions *in planta* remain unclear. Finally, various phosphatases were demonstrated to inactivate MAPK signaling through the dephosphorylation of MAPKs (Andreasson et al., 2005; Schweighofer et al., 2007; Bartels et al., 2009; Park et al., 2011; Mine et al., 2017). Nothing is known about the phosphatases inactivating MAP2Ks or MAP3Ks, if existing, or the alternative regulation processes which could be involved in the shut-down of MAPK modules. Interestingly, some MAPK related kinases have been shown to be regulated through their protein stabilization/degradation (Dóczi et al., 2007; Matsuoka et al., 2015). Similar numbers of MAPK-encoding genes are found in the genomes of other plants. Once activated, MAPKs phosphorylate protein targets. Mass-spectrometry studies have allowed for the identification of a large number of phosphopeptides fitting MAPK preferences for phosphorylation [phosphorylated Ser/Thr followed by a Pro; often referred as pT/pS-P (Songyang et al., 1996)]. For example, about one third of phosphosites identified by Rayapuram et al. (2018) were pT/pS-P sites. It is still possible that other types of kinases generated those sites either because they are not blocked by a Pro in +1 or because they have such preference. Nevertheless a number of these substrates which have been selected to contain pT/pS-Ps site have been also shown to be indeed MAPK targets (Pitzschke, 2015; Bigeard and Hirt, 2018).

### MAPK Are Involved in Various Aspects of Plant Life

Since their discovery in plants in 1980s’, MAPKs have been found to be involved in virtually all important aspects of plant life and interaction with their environment. Many review articles have discussed such functions (Colcombet and Hirt, 2008; Suarez-Rodriguez et al., 2010; Meng and Zhang, 2013; Xu and Zhang, 2015; Li et al., 2016; Chardin et al., 2017), and we will not try to be exhaustive in this section as this is not our primary objective, but we will underline selected functional questions regarding MAPK specificity.

The three Arabidopsis MAPKs -MPK3, MPK4, and MPK6-, or their homologs in other species, have been identified decades ago as strongly activated by various stresses and have been extensively characterized since. The power of genetic tools developed in Arabidopsis strongly contributed to these studies. But the main step forward comes from the identification the first PAMP (Pathogen Associated Molecular Pattern) flg22, a peptide derived from flagellum of the biotrophic pathogenic bacteria *Pseudomonas* sp., which is specifically recognized by plants as alarm signal and is a potent MAPK activator (Felix et al., 1999; Gomez-gomez and Boller, 2000; Droillard et al., 2004). In Arabidopsis, flg22 activates two functional MAPK modules: the first one is defined by MAPKKK3/5-MKK4/5-MPK3/6 and the second one by MEKK1-MKK1/2-MPK4 (Colcombet and Hirt, 2008; Suarez-Rodriguez et al., 2010; Bi et al., 2018). Beside PTI (PAMP-Triggered Immunity), MKK4/5-MPK3/6 is also activated in ETI signaling. Tsuda et al. (2013) reported the module activation upon detection of the *Pseudomonas* effector avrRPT2 by the NBS-LRR cytosolic receptor RPS2. Interestingly, activation kinetics differ largely in both cases, being rapid and transient (less than 30 min) in the case of PTI and slow and sustained (several hours) in the case of ETI. Kinases belonging to the two flg22-activated MAPK modules have been also shown to be activated upon abiotic stresses or nutritional deprivation (Ichimura et al., 2000; Teige et al., 2004; Chardin et al., 2017). Similarly, MAPK activations can be rapid and transient such as in the perception of wounding or slower and sustained. Finally, MPK3/4/6 have important functions in developmental processes. For example, MPK3/6, together with MKK4/5 and the MAP3K YODA are involved, among others, in stomatal patterning and organ abscission (Wang et al., 2007; Cho et al., 2008). Downstream of MKK7, MPK6 plays a role in shoot branching (Jia et al., 2016). MPK4, acting downstream of MKK6 (also known as ANQ) and MAP3K1/2/12 (also known as ANP1/2/3), has an important role in cytokinesis (Kosetsu et al., 2010).

The fact that the same MAPKs are involved in such distinct functions suggests that they should be able to recognize, depending of the conditions, distinct substrates, is intriguing. MPK3/6 target SPCH (SPEECHLESS) in the context of stomatal patterning (Lampard et al., 2008), PIN1 in the context of root branching (Jia et al., 2016), ICE1 in the context of freezing tolerance (Li et al., 2017; Zhao et al., 2017) and a large set of various proteins such as transcription factors and metabolic enzymes in the context of biotic stresses (for review, see Bigeard and Hirt, 2018). The phosphorylation of each substrate has not been specifically investigated in response to developmental and environmental signals. As far as we know, MAPK-dependent phosphorylation of SPCH has not been found in flg22- and biotic-response phosphoproteomics studies, whereas one of PIN1 phosphopeptides which contains a MAPK putative site was less detected in flg22-treated samples (Benschop et al., 2007). It is possible that all these MAPK substrates are phosphorylated by their cognate MAPKs regardless of the activating stimuli and that other regulatory mechanisms, acting in parallel to MAPK signaling, bring an additive ON/OFF signal necessary for the substrate function. Alternatively, another possibility is that several distinct pools of the same MAPK exist in the plant and are dedicated to act on specific substrates in specific conditions. In this scenario, two important parameters might be the subcellular localization of the kinase close to its substrate and the fact that MAPK can be, through intricate interacting processes, kept in signal-specific functional complexes. From our point of view, being able to (1) finely resolve kinase localizations, (2) distinguish protein complexes (the ensemble of MAPK interacting proteins) in which activated MAPKs are embedded depending on the activator signals and (3) describe MAPK activation in the frame of the activation of a larger signaling network are the future challenges for our understanding of MAPK cascade function in plants.

### Molecular Mechanisms of Specificity Between Kinases Involved in MAPK Modules

Mitogen activated protein kinases usually contain a conserved Thr-Glu/Asp-Tyr motif (referred as T-E/D-Y) in their activation loop which is phosphorylated on both Thr and Tyr by active MAP2Ks. The importance of surrounding amino-acids of the activation loop has not been considered so far but likely plays a role in the efficiency of phosphorylation and therefore in the specificity of MAPK activation by MAP2Ks. What is clear though, is that the interaction between MAP2Ks and MAPKs largely relies on a docking domain (D-domain) found in the N-terminal tail of MAP2Ks and a corresponding docking grove found on MAPK surface (Tanoue et al., 2000, 2001). The variation in the composition of the D-domain is largely thought to be responsible of the specificity of interactions between members of MAPK and MAP2K families. Interestingly, other proteins able to interact with MAPKs, such as phosphatases or substrates, contain a similar D-domain which has been shown to allow interaction with MAPK grooves. This model is largely supported by the resolution of crystal structures of MAPK in interaction with either partners or with peptides corresponding to D-domains (Tanoue et al., 2000, 2001). Whereas the D-domain is a disordered stretch of amino-acids, the MAPK D-groove is largely dependent on MAPK three dimensional folding and therefore on distant residues belonging to distant helixes. An additional interaction domain, named DEF (Docking site for ERK, FXFP) domain (Phe/Tyr-X-Phe/Tyr-Pro, X being any amino acids) has also been often found in MAPK substrates, usually ∼10 amino acids downstream of the phosphosite, as well as MAPK phosphatases and regulators (Fantz et al., 2001). The DEF domain binding motif, an hydrophobic grove located below kinase active site, is also know at the surface of MAPKs (Lee et al., 2004). In plants, the conservation of structure allowed the identification of the MAPK grove and some MAP2Ks present an animal/fungi-type Carboxyl-terminal domain, characterized by the sequence (Arg/Lys)_2-3_-X_2-6_-Φ-X-Φ [Φ being a hydrophobic residue (Leu, Iso, or Val)]. The functional characterization of this domain has not been performed systematically in plants. The molecular determinants of MAP3K–MAP2K interactions, which promote the MAP3K-dependent phosphorylation of conserved Ser/Thr-X_4_-Ser/Thr motifs in MAP2K activation loops, have been less well characterized and seem to be dependent on modules. For example, in the mammalian Raf-MEK-ERK2 module, the activation of MEK1 relies on a complex ballet involving allosteric interaction with the Raf-like KSR scaffold and Raf MAP3Ks (Brennan et al., 2011; Cseh et al., 2014). In plants, the fact that direct interactions have been described between MAP2Ks and MAP3Ks using yeast-2-hybrid suggested that a basic interaction is possible without the involvement of external factors such as scaffolds. But protein domains involved in MAP3K–MAP2K interactions remain unclear.

Scaffold proteins emerged in fungi and animals as important actors in MAPK signaling (Witzel et al., 2012). The first protein assuming this function, *Ste5*, has been largely characterized in *Saccharomyces cerevisiae* in the context of mating (Chol et al., 1994; Herskowitz, 1995). When a pheromone is perceived, *Ste5*, which binds the MAP3K *Ste11* and the MAP2K *Ste7* in resting conditions, recruits the MAPK *Fus3*, which, once activated, phosphorylates its substrates. Interestingly, *Fus3* also targets *Ste5* itself to negatively fine tune the module (Bhattacharyya, 2006). In yeast, other MAPK modules are used in other signaling processes, such as the filamentous growth or the osmotic stress responses. These MAPK modules, despite controlling different output events, share common kinases, and it is thought that scaffolds help to maintain signaling specificity (Chen and Thorner, 2007). In mammals, KSR (Kinase Suppressor of Ras) interacts with the MAP3K Raf, the MAP2Ks MEK1/2 and the MAPKs ERK1/2, to modulate the specificity of the ERK module and the intensity/duration of ERK activation and finally to mediate cell proliferation in response to growth factor (Nguyen et al., 2002). Whereas kinases involved in MAPK modules are easy to identify thanks to their sequence similarities, scaffold proteins belong to diverse protein families and were unknown in plants until recently. In the late 1990’s, MAP3Ks themselves were proposed to act as scaffold proteins. Indeed, Arabidopsis MEKK1 was shown to directly interact both with MKK1/2 through its kinase domain and with MPK4 through its amino-terminal tail, suggesting it could have a role in the fine tuning of MAP2K-MAPK interaction specificity (Ichimura et al., 1998). The fact that other MEKK-like MAP3Ks have generally rather long terminal tails could suggest that this tethering mechanism is conserved. To support this hypothesis, a direct interaction between MAP3K20 and MPK18 has been shown using yeast-2-hybrid and BiFC (Benhamman et al., 2017). More substantially, a plant specific scaffold protein named RACK1 (for Receptor for Activated C Kinase 1) has been recently identified as an important actor for MAPK function in response to *Pseudomonas aeruginosa* secreted protease PrpL (Cheng et al., 2015). In resting conditions, RACK1 tethers MEKK1, MKK5, and MPK3/6 in a complex anchored at the plasma membrane through its interaction with the heterotrimeric G protein. Upon PrpL perception, kinases become active and are released from the scaffold. Interestingly, RACK1 does not seem to play a role in flg22-dependent MAPK activation, suggesting that sub-populations of kinases are preconditioned to sense PrpL, thanks to their physical localization in the vicinity of G protein and the still unknown PrpL-activity receptor. To make the story more complicated, RACK1 has been shown to be involved in a number of different physiological processes besides pathogen response, and it was shown to interact with a very large diversity of plant proteins (Islas-Flores et al., 2015). Another protein that has been shown to function as a MAPK scaffold in Arabidopsis is Breaking of Asymmetry in the Stomatal Lineage (BASL). BASL binds to MPK3/6 and the MAP3K YODA to localize these proteins to the cell cortex during stomatal development (Zhang et al., 2015, 2016).

### Mechanisms for Kinase Localizations in Resting Conditions and Upon Pathway Activation

In *Arabidopsis thaliana*, MAPKs, MAP2Ks, and MAP3Ks seem to be generally composed of a kinase domain and variable tail regions which are rarely structured. Usually these kinases seem to be at least-partially extracted with a non-denaturing buffer, suggesting that they are rather soluble and not tightly bound to cellular macroscopic structures. With the possible exception of MAP3K13/14, they do not have any predictable transmembrane domains (Supplementary Table S1). Bioinformatic analysis of MAPK sequences for the presence of addressing motifs, such as NES (Nuclear Exclusion Sequences) or NLS (Nuclear Localization sequences) has been discussed previously, but their role in the signaling processes in plants has not been clearly demonstrated so far (Šamajová et al., 2013). Notably, MKK3, which is an atypical MAP2K, presents in its C-terminal tail a long NTF2 (Nuclear Transport Factor 2)-like domain which could be involved in a specific shuttling mechanism (Shibata et al., 1995; Colcombet et al., 2016). Another example is the mysterious MAP3K MEKK4 (also known as WRK18) which possesses additive domains such as Nucleotide Binding (NB)-Leucine Reach Repeat (LRR) and WRKY DNA binding domains, suggesting a function in the nucleus (Ichimura et al., 2002). Other anchoring mechanisms, such as lipidation (e.g., myristoylation or palmitoylation), have not been reported on MAPK-module related kinases, whereas they are major membrane anchoring mechanisms for plant CDPKs (Martín and Busconi, 2000; Boudsocq and Sheen, 2013). Bioinformatic tools able to predict such modifications show that MAPK-related kinases rarely present biochemical properties favorable to such modifications (Supplementary Table S1). Finally, a last localization mechanism could be the anchoring of the MAPK-related kinase through interaction with compartmentalized interacting partners.

The localization of a large number of plant MAPK-related kinases have been tentatively determined using N-terminal or C-terminal fusions with fluorescent proteins, often using transient expression systems and strong constitutive promoters (for review, see Šamajová et al., 2013; Komis et al., 2018). Overall, and despite some surprising results, these studies suggested that kinase localizations are nuclear or cytoplasmic or both. Apparently opposite results have sometimes been published. Another approach that has been used to determine kinase localization is immuno-detection using specific antibodies. As discussed previously (Šamajová et al., 2013), there are some discrepancies between the results obtained with kinases fused to fluorescent proteins and those obtained by immuno-detection of the kinase in fixed samples. Similarly, MAPK substrates are known to be localized in various compartments suggesting that at some time, the active kinase should locate in the same compartment (for review, see Bigeard and Hirt, 2018). Among others, MPK6 phosphorylates the plasma-membrane located PIN1 (Jia et al., 2016), the cytosolic ACS enzymes (Liu and Zhang, 2004), and the nuclear transcription factor ERF104 (Bethke et al., 2009). MPK3 also targets in the cytosol VIP1, a transcription factor which relocates to the nucleus once phosphorylated (Djamei et al., 2007). MPK4, in the context of cell division, targets various cytoskeletal proteins (Šamajová et al., 2013) whereas inactive MPK4 has been shown to reside in a nuclear complex with its substrate MKS1 and the transcription factor WRKY33. Activation of MPK4 by a PAMP elicitor results in phosphorylation of MKS1 and disassociation of the nuclear complex, leading to transcriptional activation of WRKY33-target genes (Qiu et al., 2008). Besides these early examples and despite the constantly growing list of identified substrates, the molecular effects of phosphorylation on substrates and where it takes place in the cell, has been poorly investigated (for review, see Pitzschke, 2015).

There are well studied examples of MAPKs in other kingdoms that are activated in the cytoplasm and subsequently accumulate in the nucleus. For example, in the *Saccharomyces* High Osmolarity Glycerol (HOG) pathway, high osmolarity does not change the cytosolic localization of either the MAP3K *Ste11* or the MAP2K *Pbs2*. In resting conditions, the MAPK *Hog1* shuttles between the cytoplasm and the nucleus but largely accumulates in the nucleus upon high osmolarity in a *Pbs2*-dependent phosphorylation manner (Ferrigno et al., 1998; Reiser et al., 1999). In mammals, the MAPK ERK2 is anchored in resting conditions in the cytosol on various docking protein such as MEK1 and KSR. Upon phosphorylation by its upstream MAP2K MEK1, ERK2 is released from its anchoring sites and an SPS motif (Asn-Pro-X-pSer/Thr-Pro-Ser) is made accessible for phosphorylation by undefined kinases. This SPS phosphorylation allows the interaction of the active ERK2 with the cargo protein importin 7 to mediate nuclear import through the nuclear pore complex (Plotnikov et al., 2011; Wortzel and Seger, 2011). In plants, it is commonly assumed that MAPK modules mediate the signal from the periphery of the cell to more internal compartments. This assumption largely relies on the fact that external stresses, and PAMPs in particular, are perceived at the plasma membrane, and a major downstream response is the modification of gene expression through the regulation of the activity of transcription factors whose function is associated with nuclei. This model is also largely influenced by what is known in other systems such as animals and yeast. The reality of the situation is, however, more complex since we know that MAPK modules can also be triggered by stimuli whose perception occurs inside the cell. For example, the detection of injected *Pseudomonas* effector AvrRpt2 by the cytosol-located RPS2 activates MKK4/5-MPK3/6 (Leister et al., 1996; Tsuda et al., 2013). Another example is MAP3K18-MKK3-MPK1/2/7/14 whose activation by the drought-related phytohormone abscisic acid (ABA) has been shown to be directly dependent on MAP3K18 protein synthesis (Boudsocq et al., 2015; Danquah et al., 2015).

Examples of the dynamic relocalization of kinases upon pathway activation by stress in plants are rather scarce. Upon treatment with ozone, activated Arabidopsis MPK3 and MPK6 were reported to be translocated to the nucleus (Ahlfors et al., 2004). A *Catharanthus roseus* MPK3 homolog was also reported to relocalyze from cytosol to nucleus within 10 min after wounding, and this relocalization was dependent of its activity (Raina et al., 2012). On the other hand, in some case MAPKs have been shown to reside in the nucleus in resting conditions, suggesting that upstream actors, likely MAP2Ks, could be the translocator of the signal from the cytosol to the nucleus. For example, MPK6 was shown to interact with ER104 in the nucleus in resting conditions (Bethke et al., 2009). Similarly, in resting conditions MPK4, MKS1 and WRKY33 define a nuclear ternary complex which dissociates upon MPK4 phosphorylation by MKK1/2 (Qiu et al., 2008). Coherently, MKK9 was indirectly shown to relocate in the nucleus to phosphorylate MPK6 upon ethylene perception in an activity dependent manner (Yoo et al., 2008). Finally, WT and constitutively active forms of MAP3K18 accumulate in protoplast nuclei whereas the kinase version mutated to be inactive (often referred as “kinase dead”) is found in the cytosol, suggesting that kinase activation triggers its relocalization to the nucleus. In this case, the MAP3K could be at least partially responsible for the movement of the signal from the cytosol to the nucleus (Mitula et al., 2015). Overall, these data are few and suggest that the subcellular localization of the kinase is not as important as it appears to be in animal systems. Possibly a majority of MAPK-related kinases shuttle between the cytosol and nucleus either freely for the smallest ones or in an energy dependent way for the larger proteins. Minimally, this trafficking does not need to be affected by the activation of the pathway to mediate a signal from cytoplasm to nucleus. Alternatively, this trafficking might be changed upon pathway activation but, because import and export process are changed the same way, the equilibrium is apparently not changed. Finally, if only a minor subpopulation of kinases is recruited by a given signal, the important shuttling mechanisms will not be detected because of the high background signal triggered by the remainder of the population.

The molecular mechanisms involved in the subcellular redistribution of MAPK-related kinases are largely unknown in plants. One recent hint concerns MPK3, whose function in response to *Botrytis cinerea*, a necrotrophic fungus triggering the gray mold disease on many plant species, has been recently shown to depend on its subcellular localization (Genenncher et al., 2016). In plants partially defective for the nucleoporin Nup88/MOS7, an important component of the nuclear pore complex (NPC), Botrytis-dependent MPK3/6 activation is strongly delayed and MPK3, but not MPK4 or MPK6, abundance is reduced whereas gene expression levels are unchanged. This reduction in protein level correlates with a decrease in MPK3 abundance in *mos7* nuclei, whereas its cytosolic level remains unchanged. Coherently, a plant expressing a version of MPK3 fused to an NES phenocopies *mos7*, becoming hypersensitive to *Botrytis*. This data shed new light on a molecular determinant of the nuclear/cytoplasmic shuttling of MAPKs and suggest that there is a connection between protein turnover and localization. These results also highlight an interesting example of differences in the behavior of the closely related MPK3 and MPK6 proteins.

## New Tools to Address Old Questions

The apparent complexity of MAPK signaling in plants is overwhelming and tools currently deployed by the plant science community clearly do not match the challenging questions regarding the internal processes of MAPK signal transduction. Recently, a number of new tools have been developed to study kinase signaling in animals which could be adapted to the study of MAPK signaling processes in plants. We believe that *in vivo* imaging, which has largely been used so far to characterize plant signaling as well as other cellular processes, is a very powerful approach that can be used to describe intimately the cellular details of MAPK signaling processes. Confocal microscopes and related techniques such as Lightsheet imaging are continuing to improve in their sensitivity and resolution, offering great potential for the study of MAPK signaling (Komis et al., 2018).

### Toward a Better Description of Dynamics in Subcellular Localization Using Confocal Microscopy

Techniques to monitor kinase subcellular localization using fluorophore fusions developed several decades ago are quite functional. They may still be improved to reduce photo-bleaching, have better resolution, etc. but overall all the technology for performing these experiments is widely available. Another strategy for imaging fluorescent biosensors with the potential to provide a less stressful experimental setup is the use of light-sheet microscopy (Maizel et al., 2011). The light-sheet approach exposes the sample to much lower amounts of excitation energy during imaging experiments, which allows for more prolonged time-series that do not risk photo-damage to the sample. This approach can be particularly useful for studying growth and development of root tissue and studying cell division. Because MAPK signaling has been shown to play a role in regulating cytokinesis (Beck et al., 2010, 2011; Kosetsu et al., 2010), it may be possible to use this approach to probe the spatial and temporal dynamics of these MAPK pathways in this important process.

We believe that efforts to gain a better understanding of kinase localization in resting conditions and upon activation by stresses should largely concentrate on the generation of accurate biological material. Optimally, to avoid artifacts in the localization using fusion with fluorescent markers, constructs driving the expression of chimeric kinases should retain as many of the putative regulatory sequences as possible from the native gene which could impact the final quantity of transcript and therefore of protein. This includes promoters, Untranslated Translated Regions (UTRs), introns and terminators. As the fluorescent tag may also induce artifacts in the localization or function of the kinase, a wise choice would be to rescue the phenotype conferred by loss-of-function recessive mutations of the corresponding gene. Finally, as T-DNA insertions often lead to variable levels of expression, a final criterion would be to choose lines for which expression is tested at the transcript and/or protein level using specific antibodies. To our knowledge, this type of extensive characterization of MAPK reporter lines has not been reported so far.

If it is indeed the case that activation-induced kinase relocalization is less dramatic in plant than in animal systems, the velocity of nuclear-cytoplasmic shuttling may be an important parameter to measure if we want to understand finely the signal transduction processes within the cell. As mentioned, shuttling turnover might increase upon activation without being detectable in the ratio between nuclear and cytoplasmic kinase. Fluorescence recovery after photobleaching (FRAP) experiments may be used to access such kinetic variables. In human cells, it has been successfully used to characterize EGF-induced specific ERK2 release from cytoplasmic anchor and its subsequent nuclear translocation (Burack and Shaw, 2005). In Arabidopsis, a very elegant study showed using FRAP that MPK6 nuclear signaling is reinforced after the asymmetrical division of the MMC (Meristemoid Mother Cell) to form the SLGC (stomatal lineage ground cell) and the meristemoid in order to phosphorylate the nuclear master regulator SPCH during the process of stomatal patterning (Zhang et al., 2016). Finally, a last very challenging question is to be able to distinguish sub-populations of proteins on the basis of their phosphorylation. For example, it would be very useful to identify among a whole MAPK population, the localization of the fraction having phosphorylated T-E/D-Y motifs (e.g., being activated) upon various developmental or environmental signals. A potential approach could be to perform FRET [Förster Resonance Energy Transfer or Fluorescence Resonance Energy Transfer (Hink et al., 2002)] experiments on fixed samples using a pair of antibodies coupled to a pair of FRET fluorophores, the first one recognizing a specific MAPK and the other one the phosphorylated MAPK activation loop motif pT-E/D-pY. FRET refers to the biophysical mechanism of energy transfer between two light-sensitive molecules (fluorophores) which are close enough, this proximity being detected optically. If a MAPK is phosphorylated and therefore labeled by the two antibodies, the proximity of the fluorophore may be detected through the FRET effect. At the cellular level it could provide a good indication of the location of MAPK activation.

### Deciphering the Cellular Localization of MAPK Interactions

BiFC (Bimolecular Fluorescence Complementation) has been widely used to test kinase interactions in plants. This technique also gives access to the spatial distribution of these interactions. Nevertheless, as for the study of kinase subcellular localization discussed above, it is largely coupled with very high level expression of the chimeric proteins as well as the use of transient expression systems. For example, agro-infiltration of *Nicotiana benthamiana* leaves is a very simple and popular technique which usually involves ORF expression under the control of strong promoters and is a transient expression assay. In addition, because the BiFC interaction is thought to be effectively irreversible, this approach does not allow one to observe changes in protein–protein interaction over time or in response to a treatment, and the method may also induce some/many false positives. It would be of considerable interest to be able to observe dynamic interactions between MAPK-cascade proteins that change in response to different stimuli. For this type of experiment, using FRET or FRET-Fluorescence-Lifetime Imaging Microscopy (FLIM) approach could be more productive. As a successful but up to now unique example in plants, it has been shown using FRET that Arabidopsis MPK6 forms a complex with Ethylene Response Factor 104 (ERF104) in the nucleus and that this complex is disrupted when cells are treated with the PAMP flg22 (Bethke et al., 2009). Recent work involving transcription factors that regulate cell fate specification in roots has demonstrated how FRET-FLIM can be a powerful tool for visualizing dynamic protein–protein interactions in living tissues (Long et al., 2017). Application of these methods to the study of protein-protein interactions among MAPK-related proteins has the potential to reveal important new information regarding the spatial regulation of kinase associations.

### MAPK Activity Sensors to Study Subcellular and Cell-Type Localization of MAPK Module Activity

In addition to characterizing the dynamic localizations and interactions of MAPK cascade proteins, it is also of interest to be able to measure the activity of these kinases in living plants. Indeed, up to now plant MAPK activities have been measured using coarse biochemical techniques such as immunoprecipitation followed by *in vitro* phosphorylation of substrates, *in gel* kinase assays and detection of phosphorylated (i.e., activated) MAPK forms by Western Blot. Together, all these techniques have provided a robust base of knowledge of MAPK signaling but, because they required a large starting amount of biological material, they generally are applied on whole plant/plantlets or at best on large organs (i.e., root, shoot, etc.). To investigate MAPK activity in very specific cell types, it would be necessary to develop either techniques based on cell sorting, which are likely difficult to setup to measure the transient activation of stress-responsive kinases, or non-invasive techniques based on activity sensors. A typical example of this problematic is the measurement of MAPK activity in stomatal guard cells which is rarely been done for technical reason despite the genetic studies that have suggested they play major roles (Jammes et al., 2011; Montillet et al., 2013; Jakobson et al., 2016). Another level of investigation is the cellular compartmentalization of kinase activation. Indeed, as MAPK localization is suspected to be involved in the specificity of responses triggered by different signals, it could be important to develop ways to characterize sub-populations of MAPKs based on their spatial organization in the cell. In plants, strategies using immunolocalization of phosphorylated (and therefore active) forms of MAPKs within plant tissue have been reported. In some cases, antibodies have been developed against the phosphorylated activation loop of a plant MAPK (Šamaj et al., 2002; Ovecka et al., 2014). More often, an antibody raised against the mammalian phosphoERK2 has been adapted in planta but, being not specific of any MAPK in particular, a comparison with mapk deficient plants is necessary to confirm the specificity of signal (Beck et al., 2011; Kohoutová et al., 2015). This approach gave interesting results, particularly in the context of the root for which the protocol of fixing and immunolocalization are well established.

**FIGURE 1:**
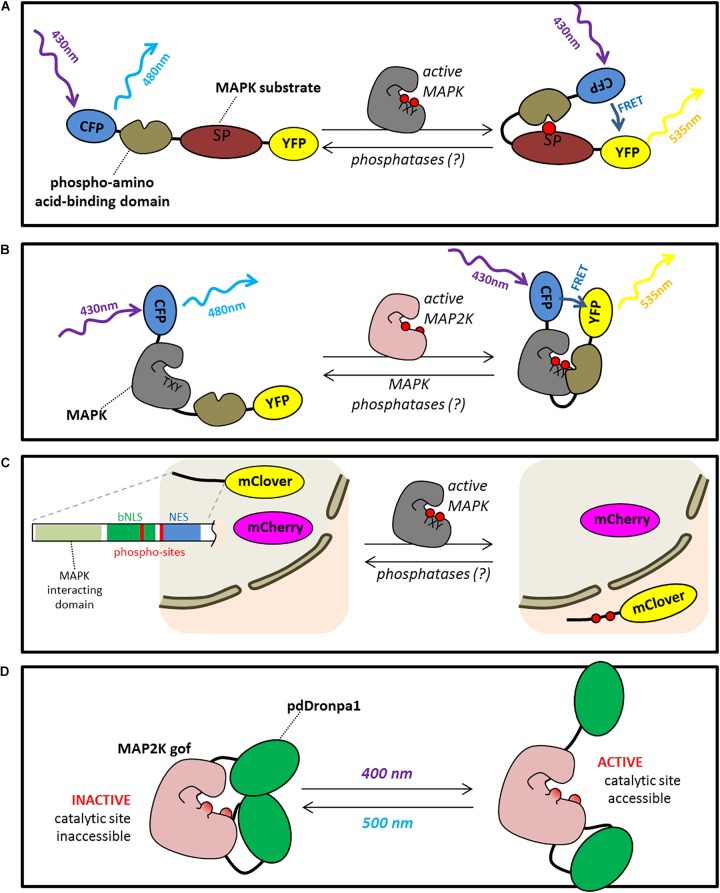
Microcopy based tools to study and manipulate MAPK signaling. **(A)** Substrate based activity sensors (SBAS) are constituted of a pair of fluorescent proteins able to perform FRET (for example YFP and CFP) separated by a phosphosite-containing peptide derived from a kinase substrate and a phospho-amino acid-binding domain. Upon phosphorylation of the substrate domain, the sensor undergoes a conformational change triggering a FRET effect. **(B)** Probes for conformation changes (PCC) are constituted of a pair of fluorescent proteins able to perform FRET separated by a kinase which, upon activation, undergoes conformational changes triggering a FRET effect. **(C)** Kinase translocation reporters (KTRs) is composed of a fluorophore whose localization is driven by a NES/NLS sequence carrying a MAPK phosphosite. Upon phosphorylation, KTR relocalizes in the nucleus. An additive fluorophore is co-expressed in the cell allowing a ratiometric quantification of the relocalization. **(D)** Photo-switchable kinases is composed of a constitutive active kinase (for example, a MAP2K carrying 2 phospho-mimicking mutations on the residues which are phosphorylated by upstream MAP3Ks) which is neutralized by two dimerising pdDROMPA domains. Upon illumination at 500 nm pdDROMPAs dissociate and the kinase active site becomes accessible able to phosphorylate downstream targets (ON). This process is reversible by using an illumination at 400 nm (OFF).

One alternative strategy, which could be used to monitor kinase activity at both tissue and sub-cellular levels would be to use fluorescence-based genetically encoded biosensors. Such biosensors have been now largely developed for use in animal cells, allowing easy activity monitoring using microscopy (Oldach and Zhang, 2014). These sensors are usually based on kinase activity-triggered internal FRET between two fluorescent protein domains present on the sensor (Figure 1). It should be possible to adapt these sensors for use in plants, although to date there have been no published reports of this in plants. Roughly, established biosensors for kinase activity fall into two categories: Substrate Based Activity Sensors (SBAS) and Probes for Conformation Changes (PCC). SBAS are usually constituted of a pair of fluorescent proteins able to perform FRET separated by a phosphosite-containing peptide derived from a kinase substrate and a phospho-amino acid-binding domain (Figure 1A). In PCCs, the fluorescent proteins are separated by a kinase or a kinase followed by phospho-amino acid-binding domain (Figure 1B). Upon kinase activation/substrate phosphorylation, a conformation change is detected through intramolecular variation of the FRET effect. These two approaches have been used for various kinases in non-plant systems [reviewed in (Oldach and Zhang, 2014)]. The first reports of SBAS probes specific for MAPKs include ERKUS (Sato et al., 2002) and EKAR (Harvey et al., 2008). Over the years, improved versions of these sensors have been reported, such as EKAREV (Komatsu et al., 2011). The EKAR and ERKUS sensors contain ERK substrate domains separated from phospho-amino acid binding domains by flexible linkers. Phosphorylation of the sensor by MAPK causes an increase in the FRET of these sensors by bringing the CFP and YFP domains into closer proximity. A common theme with these FRET sensors is that their design and optimization is empirical and time consuming. Authors usually report the testing of many variants with various combinations of domains and lengths of linkers to reach the best signal/noise ratio and a dynamic response curve. Additionally, if theoretically such sensors could provide resolution at the whole cell or tissue level, there often exist versions containing an additive localization signal (NES/NLS) to more easily monitor signaling in specific compartments. In plants, one limitation to using SBASs is that many known substrates are often not specific for a single MAPK (or cross-phosphorylation by other MAPKs has not been tested) and therefore the probe may report the activities of multiple MAPK modules active in parallel, making the picture blurry. This problem is enhanced by the fact that sensors are usually expressed under the control of a strong promoter to reach a comfortable fluorescent signal. Consequently, once sensor lines are established, it would be necessary to validate the sensor specificity by crossing with mutants impaired in MAPK suspected to be involved in the process. On the other hand, the fact that these probes may report the activity of more than one kinase could also be seen as reflecting the reality of the signaling process happening in the cell. If multiple MAPKs are able to phosphorylate a given substrate, it may be quite valuable to be able to measure the integrated sum of the kinase activities that contribute to that substrate’s phosphorylation status in order to build a better understanding of the overall signaling network operating within the cell.

Probes for conformation changes for MAPK activity have not been as widely deployed as SBAS probes, but an example of one for reporting ERK activity in mammalian cells is Miu2 (Fujioka et al., 2006). The Miu2 sensor consists of the MAPK ERK sandwiched between CFP and YFP domains. Binding of MEK to the sensor results in a modest decrease in FRET, which can be used to infer activation of ERK. Interpretation of the results obtained with this type of sensor is complicated by the fact that the FRET change is tied to binding of MEK, so that activated ERK that has dissociated from MEK will not show the FRET change. It therefore serves as a direct reporter for MEK-ERK interaction and an indirect reporter of ERK activity. Using PCCs build with both a MAPK and a phospho-amino acid binding domain should be more efficient. One of the theoretical advantages of PCCs is that it is specific of a single kinase.

In addition to the FRET-based sensors discussed above, another potential strategy for a genetically encoded sensor of MAP kinase activity would be to work with circularly permuted versions of YFP. This approach was used to produce the Pericam calcium sensors (Nagai et al., 2001). Briefly, a circularly permuted version of YFP is engineered by swapping the amino and carboxyl YFP portions which are reconnected by a short spacer between the original termini, and then fused with the Ca^2+^-binding protein calmodulin and its target peptide M13 tails such that binding of calcium by the protein causes YFP fluorescence to either increase or decrease substantially, depending on which version of Pericam is used (Nagai et al., 2001). It has been subsequently shown that swapping different tails onto this type of sensor can make it responsive to different signaling events. For example, Kupinski and coworkers developed Smo-IP, a fluorescent sensor that reports the phosphorylation status of the Drosophila Smoothened protein by modifying the Inverse-pericam calcium sensor (Nagai et al., 2001; Kupinski et al., 2013). It is possible that adding the correct tails to one of the Pericam sensors could produce a MAPK reporter in which phosphorylation produced the conformational changes needed to modify fluorescence, although this has not yet been reported in the literature.

An alternative, elegant design for kinase sensors is that of the kinase translocation reporters (KTRs) (Regot et al., 2014). In KTRs, which only have one fluorophore, phosphorylation modifies a nuclear localization/nuclear export domain such that the localization of the reporter shifts from the nucleus to the cytoplasm (Figure 1). Kinase activation can therefore be measured by determining the ratio of nuclear to cytoplasmic fluorescence of the reporter. In the original report, specific KTRs were designed for the 3 main animal MAPKs, ERK, p38 and JNK and used to characterize MAPK signaling in a fibroblast cell culture (Regot et al., 2014). A limitation of such a tool is obviously that it does not provide sub-cellular resolution, but a recent publication presented the use of KTRs to monitor MAPK signaling at the whole organism level using living *Caenorhabditis elegans* (de la Cova et al., 2017). This new version of the KTR strategy involved expressing a bicistronic transcript that encodes the KTR as well as an RFP control protein that is constitutively localized to the nucleus to provide an internal control for more accurately measuring the abundance of GFP-tagged KTR protein in the nucleus. This approach could be particularly useful in plants where quantitating the total fluorescent signal in the cytoplasm versus nucleus in intact tissue can be challenging due to the large central vacuole present in the cells and their complex geometry. The same considerations regarding substrate specificity discussed above for SBASs would also apply to KTRs. In addition, it will be necessary to determine if the NES/NLS sequence used for the KTR sensors in animals drives the same phosphorylation-dependent changes in localization in plant cells.

The FRET-based reporters have been used to measure the activity of MAPKs in animal cell cultures, *C. elegans* worms, and transgenic mice (Kamioka et al., 2012; Tomida et al., 2012; Aoki et al., 2013; Ryu et al., 2015). The FRET sensors used in these studies have allowed the authors to characterize the amplitude and duration of kinase activation in response to various input stimuli. One would expect that this same approach could be applied to plants by modifying these sensors to respond to plant MAPKs. The success of FRET sensors for studying the dynamics of sugar, calcium, pH, ABA, ATP, and gibberellic acid (GA) levels in plants further supports this expectation. As sensors for reporting MAPK activity are developed, it will be important for the research community to adapt methods for validating the specificity and dynamic range of those sensors.

Another approach to investigating the subcellular dynamics of MAPK activation is to use antibodies that are specific to the activated form of the kinase to perform immuno-cytochemistry on fixed tissue samples collected before and after treatment with a stimulus known to activate MAP kinases. This approach has been used to show that activation of the MAPK SIMK by salt stress in *Medicago sativa* roots results in accumulation of the active kinase in the cytoplasm (Ovecka et al., 2014).

### Playing at Will With MAPK Activity *in vivo*

Besides genetic analyses of specific knock-out (KO) mutants, very interesting tools aiming to manipulate MAPK activity *in planta* have also emerged. For example, plants in which a constitutively active (CA) MAPK replaces the endogenous one provided an interesting alternative material in which the identification of MAPK-controlled mechanisms is theoretically easier (Berriri et al., 2012; Genot et al., 2017). Nevertheless, because MAPK signaling is quite dynamic, controls multiple aspects of plant physiology and development, and triggers cascades of processes, it is often difficult to distinguish in the complex KO/CA phenotype MAPK direct responses from events which are secondary consequences of this direct response.

Research now has the need for tools that allow one to rapidly activate/inactivate MAPK cascades in plants having otherwise WT phenotypes. Such tools have been established for MPK3, MPK4, and MPK6 using a chemical-genetic approach in which one engineers an inhibitor-sensitive version of the kinase and uses it to replace the wild-type (Bishop et al., 2000; Brodersen et al., 2006; Xu et al., 2014, 2016; Leissing et al., 2016; Su et al., 2017). The inhibitor used in these studies, Na-PP1, is a bulky derivative of ATP that does not bind to the ATP binding pocket of wild-type kinases, but will bind to a kinase if a single, specific amino acid substitution is made (Bishop et al., 2000). Treating a plant with Na-PP1 will thereby selectively switch off the activity of the mutant kinase. In the absence of the inhibitor, the mutant kinase retains its wild-type activity. This chemical genetic approach to manipulating plant MAPKs was first described for MPK4 (Brodersen et al., 2006), where it was shown that adding the inhibitor triggered the activation of defense-response genes, mimicking what is observed for the *mpk4* loss-of-function mutant. In the case of MPK3 and MPK6, this inhibitor-sensitive kinase approach was particularly helpful because the *mpk3mpk6* double-mutant combination produces a very strong developmental phenotype that makes stress-response phenotyping impossible. *mpk3mpk6* plants rescued by an Na-PPi sensitive version of MPK3 or MPK6 have a WT phenotype, but the use the Na-PPi blocker allows the total shut down of the pathway (Xu et al., 2014, 2016; Su et al., 2017, 2018). Up to now, this tool has mainly be used to generate biological material impaired in MAPK activity in order to validate downstream targets, but it also has the potential to provide a powerful method for dynamically impairing MAPK signaling. For example, one could apply the drug at particular stage of epidermal development to help decipher the function of the MAPK-BASL module in stomatal patterning process. Coupled with fluorescent reporters and live-cell imaging, the use of this type of chemical genetics could allow a researcher to specifically perturb and monitor signaling with unprecedented precision.

Another very promising tool is the use of genetically encoded inhibitors of specific kinases. One possibility is to overexpress modified interacting proteins which will inhibit signal transduction by competing with the native interactor. In theory, the simple expression of D-domains (found in MAPK substrates or upstream MAP2Ks) in cells should inhibit MAPKs by occupying the docking grove of the kinase. To the best of our knowledge, this approach has not yet been tested. Alternatively, genetically encoded antibodies may be used to target a particular protein. For example, the role of AUXIN BINDING PROTEIN1 (ABP1) in cell cycle regulation has been investigated using the expression of a single-chain variable fragment (scFv) made from the fusion of the two hypervariable regions of the heavy and light chains of an anti-ABP1 monoclonal antibody previously shown to block the activity of ABP1 (David et al., 2007). Up until recently, the time necessary to develop such tools built from conventional antibodies made this approach almost impossible. Nevertheless, the recent identification of single chain antibodies in Camelids has allowed the easy development of small (∼15 KD) antigen-binding fragments (also known as nanobodies) which may be expressed from the nucleus. Several examples of *in vivo* manipulation of enzyme activities in mammalian cells may be found in the literature [for a review see (Beghein and Gettemans, 2017)]. It may be proposed that, with an appropriate tag, the expression of such domains could allow for the inactivation of compartment-specific populations of MAPKs to investigate their role in plant physiology and response to stresses.

The genetic approaches described above provide tools for switching off a kinase. It would also be helpful to be able to rapidly switch the pathway on, but the corresponding chemically switchable “gain-of-function” mutants are not available and the strategy to reach this goal less obvious. Nevertheless in animal systems a very promising tool has been published recently (Zhou et al., 2017) (Figure 1D). It consists of photo-switchable versions of kinases that can be converted from an inactive to active state by illumination with specific wavelengths of visible light. The basic strategy is to take a constitutively active version of a kinase and insert a version of the photo-switchable fluorescent protein DRONPA into the protein sequence at a specific structural position. The result is a kinase whose activity can be toggled on and off by illuminating with 400 nm versus 500 nm light. Because constitutively active versions of plant MAP2Ks have been well-described, it should be possible to engineer a photo-switchable plant MAP2K. An obvious challenge for moving this system from animal cells to plants is the simple fact that plants need to grow in the light. There are many strategies that one could imagine using to overcome this challenge. First of all, transient protoplast experiments can be performed in which the cells are maintained in the dark, which would allow for photo-control of the kinase activity without confounding effects of light from the growth environment. Alternatively, some suspension cell culture grow well in the dark when fed with a rich medium. Secondly, if one wanted to work with this system in a whole plant system, it may be possible to use LED lights or wavelength filters to remove light in the 400 nm range that activates the kinase during the process of developing the transgenic lines. The default state of the photoswitchable kinase in the dark is “off,” so as long as light in the 400 nm range is not present, it should remain off. The challenge would be to develop growth conditions where relatively healthy plants could be produced that did not trigger constitutive activation of the kinase. If such a tool could be developed, it would allow the researcher to target spatially and temporally the activation of a MAPK pathway in an unprecedented manner.

### Sample Mounting and Manipulation

Because MAPK pathways are activated by a wide range of stresses, cautious handling of living samples is particularly crucial for this type of analysis. For this reason, mock controls need to be systematically and very seriously done when working on stress-activated MAPKs and particularly when using live-cell imaging methods. Samples should be handled as gently as possible and equilibrate as long as possible in the experimental conditions used for imaging under the microscope.

One of the challenges of live-cell imaging studies is to create an experimental system mimicking environment that is as close to “natural” as possible. For studying MAPK signaling in plants, one would like to be able to observe living seedlings or tissue under the confocal microscope in such a manner that stress pathways are not constitutively activated. In addition, it would be desirable in many cases to be able to apply discreet stress treatments to the sample during the imaging process. An elegant solution to this challenge for live-cell imaging of root tissue is a microfluidic device called the Root Chip (Grossmann et al., 2011; Stanley et al., 2018). Using this system, the roots of Arabidopsis seedlings grow through narrow channels along the surface of a coverslip. Precise control of the fluid flowing through the channels is achieved by microfluidic control, which allows the researcher to perform pulse-chase experiments to test the effects of different chemical treatments on roots during imaging. Light sheet microscopy offers an attractive alternative to confocal microscopy for visualizing fluorescent probes. Indeed, recent work has shown the utility of light sheet microscopy for dynamically tracking the subcellular localization of MAPKs and a substrate in Arabidopsis (Smékalová et al., 2014; Komis et al., 2018; Ovečka et al., 2018).

## Conclusion

In this review we have explored our current understanding of how MAPK proteins are organized at the subcellular level. It is clear from this analysis that we are at a very early stage in the process of building a comprehensive understanding of what role MAPK protein localization plays in pathway function. For example, activation of the FLS2 receptor by flg22 binding initiates a signaling pathway that activates a MAPK cascade in which MPK6 ultimately phosphorylates a number of target substrates localized in the nucleus. It is currently not known, however, what the mobile elements of this signaling pathway are that allow this information to travel from the receptor at the plasma membrane to the nucleus. Application of the different live cell imaging methods discussed in this review has the potential to shed new light on how MAPK activation affects protein localization, movement, and function. The ultimate goal would be to build a mechanistic model to explain the signaling pathway that incorporates the activity status of the kinases and their subcellular localizations and how these change in response to pathway activation and deactivation.

## Author Contributions

All authors listed have made a substantial, direct and intellectual contribution to the work, and approved it for publication.

## Conflict of Interest Statement

The authors declare that the research was conducted in the absence of any commercial or financial relationships that could be construed as a potential conflict of interest.
